# Temporal modelling of *Lymnaea natalensis* (Krauss, 1848) in tropical aquatic habitats

**DOI:** 10.4102/ojvr.v90i1.2023

**Published:** 2023-05-10

**Authors:** Opeyemi G. Oso, Joseph O. Sunday, Alexander B. Odaibo

**Affiliations:** 1 Department of Zoology, Faculty of Science, University of Ibadan, Ibadan, Nigeria; 2 Parasitology Unit, Department of Zoology, Kwara State University, Malete, Kwara State, Nigeria

**Keywords:** geographic information system (GIS/RS), modelling, risk map, *Lymnaea natalensis* (*L. natalensis*), rain forest

## Abstract

*Lymnaea natalensis* is the only snail intermediate host of *Fasciola gigantica*, the causative agent of fascioliasis, in Nigeria. The species also serves as intermediate host for many other African trematode species of medical and veterinary importance, and it is found throughout the country. However, there is no detailed information on the factors that influence its distribution and seasonal abundance in the tropical aquatic habitats in Nigeria. This study used the geographic information system and remotely sensed data to develop models for predicting the distribution of *L. natalensis* in South-Western Nigeria. Both land surface temperature (LST) and normalised difference vegetation index (NDVI) were extracted from Landsat satellite imagery; other variables (slope and elevation) were extracted from a digital elevation model (DEM) while rainfall data were retrieved from the European Meteorology Research Programme (EMRP). These environmental variables were integrated into a geographic information system (GIS) to predict suitable habitats of *L. natalensis* using exploratory regression. A total of 1410 *L. natalensis* snails were collected *vis-à-vis* 22 sampling sites. Built-up areas recorded more *L. natalensis* compared with farmlands. There was no significant difference in the abundance of snails with season (*p* > 0.05). The regression models showed that rainfall, NDVI, and slope were predictors of *L. natalensis* distribution. The habitats suitable for *L. natalensis* were central areas, while areas to the north and south were not suitable for *L. natalensis*.

**Contribution:**

The predictive risk models of *L. natalensis* in the study will be useful in mapping other areas where the snail sampling could not be conducted.

## Introduction

Lymnaeid snails act as intermediate hosts of medically and veterinary important digenean trematodes, which are of public health importance in the tropics and sub-tropical areas (Chongmobmi & Panda 2018).

Generally, digenetic trematodes are multi-host parasites that infect freshwater snails, marine snails, and vertebrates during their intermediate life stages and adult stages, respectively. *Lymnaea natalensis* is of major importance in the transmission of fascioliasis in many parts of tropical and sub-tropical countries (Atwa & Bakry 2019). Fascioliasis is endemic in several regions across the world and it is a serious infectious parasitic disease of domestic ruminants and humans (Haridy et al. 2002). It is caused by liver flukes belonging to genus *Fasciola*, of which *Fasciola gigantica* and *Fasciola hepatica* are the two main species (Chiejina 1994; Halakou et al. 2017). Reports show that *F. hepatica* is endemic in Europe, the Americas, Oceania, Africa and Asia while *F. gigantica* has a focal distribution in Africa and Asia (Ashrafi et al. 2006; Kasahara et al. 2021; Le et al. 2008; Moghaddam et al. 2004; Seid & Melese 2018; Valero et al. 2009). Various animals, such as sheep, goats, cattle, buffalos, horses, donkeys, camels and rabbits, have shown very high infection rates in different areas (Farag 1998; Karshima, Bata & Bobbo 2016; Peterson & Barnes 2020). Initially, human infection with fascioliasis was very sporadic; however, in recent times, clinical cases and outbreaks were reported (Haseeb et al. 2002). Globally, more than 2.4 million people are infected with fascioliasis while approximately 180 million people are at risk of the infection (World Health Organization [WHO] 2007). Hence, an increase in *L. natalensis* density often leads to high prevalence of trematode infection in different ecological zones (Patz et al. 2000; Smith 2001). Various processes of environmental degradation result in the spread of diseases caused by digenetic trematodes through *L. natalensis* (Johnson & Chase 2004). Such environmental degradation (e.g., deforestation, dam construction etc.) creates suitable habitats (e.g., pool, dam), where different snail species can survive.

The distribution and abundance of *L. natalensis* is highly related to certain known environmental conditions (Dutra et al. 2010; Olaechea 1994; Yigezu et al. 2018) and this conforms to the concept of natural nidality of disease and landscape epidemiology (Pavlovsky 1966). Ecological factors (temperature, light, pH, depth of water, vegetation, soil chemical composition, competitive snail population, and water current) in particular have been reported to be a sensitive means of forecasting annual abundance of *L. natalensis* and their variability (Fuentes 2006; Malone & Yilma 1999; Soliman 2008). Infection in humans is not absolutely limited to areas where fascioliasis is predominant in animals; however, faeces from infected persons can maintain transmission for prolonged periods, most especially where open defaecation is often practised (WHO 2007). The practice of nomadic animal farming is common in the study areas (Yewa North Local Government Area [YNLGA] of Ogun State, South-Western Nigeria); hence, animal movement is often seen throughout the year. Nomadic farmers bring their cattle from the north to the southern parts of the country; cattle are found in large quantities during the dry season because of the green vegetation that is available almost throughout the year in the south. As a result of the absence of restrictions in animal importation from Niger and other Northern Nigeria to the south for grazing purposes, movement of infected animals into an uninfected area cannot be overemphasised. In preparation for adequate planning for successful intervention in the control of parasitic diseases, people living in at risk areas are often targeted for optimum control strategy; hence, current spatial distribution of parasites and intermediate host species is often considered. In most cases, during an outbreak of diseases, more funds are often required in the field for epidemiological survey, technical expertise, parasite determination and confirmation, which could be time consuming. Therefore, provision of geographical modelling could serve as a good alternative, which is cheaper and apt in providing necessary information for policymakers. Geo-information provides effective monitoring, prevention, control and management of diseases that are of veterinary and medical importance in the world (Arjkumpa et al. 2020; Gavin 2002). In most cases, the use of mapping facilitates and promotes creative problem solving and sound decision-making with lasting positive impacts on human lives (Gavin 2002). Most of the attention on the geographical modelling of gastropods has focused on the family Planorbidae, which includes freshwater snails that transmit human schistosomes (Deka 2022; Fasona et al. 2021; Mahmoud et al. 2022). Very limited attention has been given to the family Lymnaeidae, which also transmit parasites of medical and veterinary importance in Nigeria. Therefore, this study aims to examine the spatial distribution of *Lymnaea natalensis* in trematodes infested areas and to identify suitable and unsuitable areas for the survival of the species.

## Materials and methods

### Study area

Yewa River is located in Yewa North Local Government Area of Ogun State, South-Western Nigeria; it is close to the Republic of Benin. The river is divided into more than 10 tributaries in the Local Government Area (LGA) (latitudes 6^o^52′08″N–7^o^25′28″N and longitudes 2^o^43′09″E–3^o^07′13″E). Each tributary is given a specific name in different villages while some villages retain the name as Yewa River. Snail sampling was carried out in farmland and built-up areas. The major difference between built-up areas and farmland is the population of human dwellers. Built-up areas had more population of human dwellers compared with the farmland. Rearing of cattle and crop farming are the major occupations of residents in the LGA. Nomadic cattle rearing is practised all over the LGA because of the large land mass and green vegetation that is peculiar to the zone throughout the year. Informed consent was obtained from participants, while permission to carry out the study was obtained from the village heads and state ministry of health before the commencement of the study.

**FIGURE 1:**
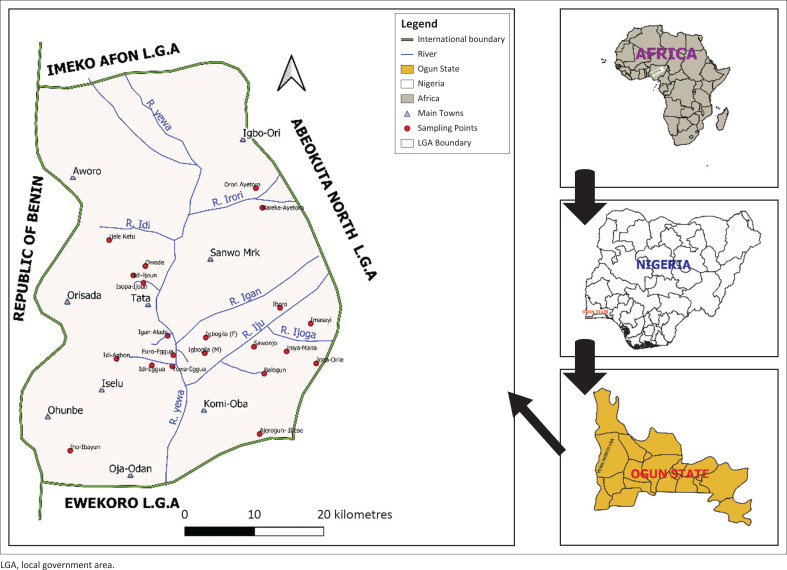
Map of study area.

Sampling station selection was based on water contact sites, representing known habitats of *L. natalensis* (Figure 1). Twenty-two sampling stations were purposively selected, and snail sampling was conducted in each site, using the scooping method for 24 months (Coulibaly & Madsen 1990). Snails collected were identified according to identification guides provided by Brown and Kristensen (1989), and to establish the seasonal breeding trends of the snails, the shell morphometrics (length [L], width [W], aperture length [AL], and aperture width [AW]) of each snail was measured with the aid of vernier caliper (Brown & Kristensen 1989). Trematode infection in snails was assessed by exposing each snail, placed inside a petri dish containing distilled water, to sunlight to shed cercariae and by crushing method (Okoli & Odaibo 1999). Relative humidity data were collected from Ogun State Meteorological Agency. Rainfall data were downloaded from the European Meteorology Research Program (http://ecmwf.int). Landsat 8 was downloaded from United States Geological Survey (USGS) Earth Explorer website; band 4 and band 5 were used to generate normalised difference vegetation index (NDVI) while elevation and slope were derived from digital elevation model of the Advanced Spaceborne Thermal Emission Radiometer (ASTER) (https://www.nasa.gov). For land surface temperature (LST), thermal band of the operational landsat imager (OLI) sensor was used for calculating top of atmospheric (TOA) spectral radiance, proportion of vegetation, and emissivity before arriving at the final LST in degree Celsius. Independent t-test was used to determine significant differences in seasonal snail density, while exploratory regression was used for geospatial data.

### Ethical considerations

Approval to conduct the study was approved by the Kwara State Ministry of Health (ref: MOH/KS/RERC/777/58).

## Results

### Distribution and abundance of Lymnaea natalensis

**FIGURE 2:**
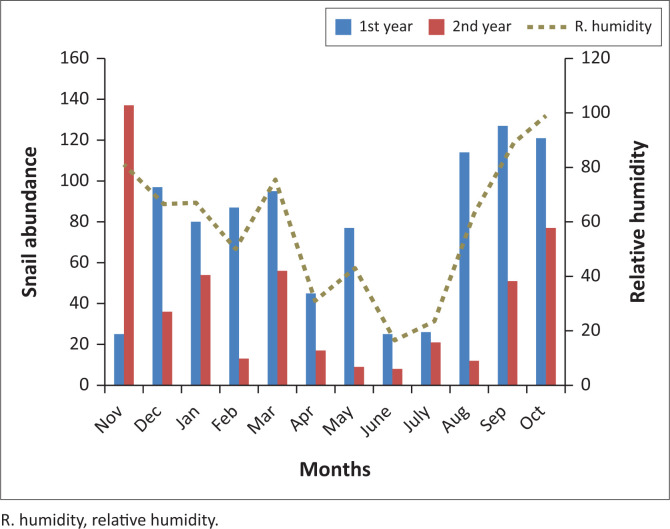
Monthly variations in the abundance of *Lymnaea natalensis* in relation to relative humidity.

**FIGURE 3:**
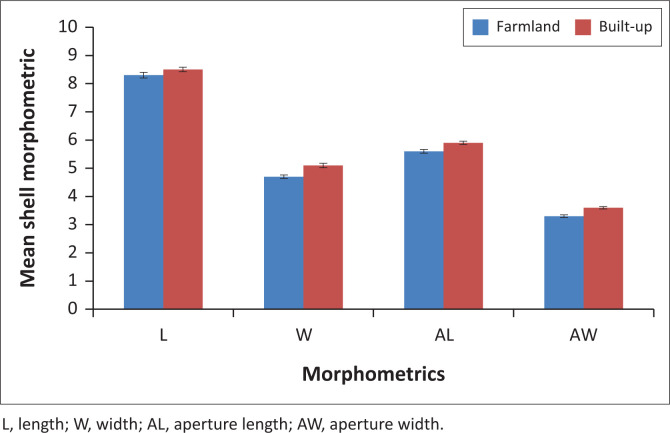
Morphometric of *Lymnaea natalensis* in Yewa North local government area.

*Lymnaea natalensis* occurred throughout the dry and rainy seasons during the study. A total of 1410 *L. natalensis* were collected, in which the first sampling year yielded more snails compared with the following sampling year. Relative humidity varied from 84% to 99% (Figure 2). The highest abundance of the snail was recovered from Iju River in Ijale Ketu. There was no significant difference (*p* > 0.05) in the snail abundance with season. The mean shell length of *L. natalensis* was 8.6 ± 2.7 mm (Figure 3). The highest abundance of snail collected was recorded in September of first sampling year, while the least was recorded in June of the same year. During the second sampling year, the highest snail abundance was recorded in October while the least was recovered in May (Table 1). Built-up areas harboured more snails compared with the farmland (Table 2). There were no infections recorded in the snail species during the sampling period.

**TABLE 1:** Monthly distribution of *L. natalensis* collected in built-up and farmland sampling stations.

Location description	Month	1st year	2nd year	Total
Built-up	Nov.	10	79	89
	Dec.	71	36	107
	Jan.	43	43	86
	Feb.	48	12	60
	Mar.	71	54	125
	April	31	15	46
	May	68	7	75
	June	4	8	12
	July	10	3	13
	Aug.	96	12	108
	Sep.	122	37	159
	Oct.	88	19	107
Farmland	Nov.	15	58	73
	Dec.	26	0	26
	Jan.	37	11	48
	Feb.	39	1	40
	Mar.	24	2	26
	April	14	2	16
	May	9	2	11
	June	21	0	21
	July	16	18	34
	Aug.	18	0	18
	Sep.	5	14	19
	Oct.	33	58	91

**TABLE 2:** Variations in shell length (X ± s.d.) of *Lymnaea natalensis* in different seasons from built-up and farmland.

Types of study location	Number of study sites	1st year				2nd year			
		Rainy season		Dry season		Rainy season		Dry season	
		*n*	X ± s.d.	*n*	X ± s.d.	*n*	X ± s.d.	*n*	X ± s.d.
Built-up	10	419	8.3 ± 2.2†	243	9.8 ± 4.9†	101	8.7 ± 2.3†	224	8.7 ± 2.1†
Farmland	11	116	7.5 ± 2.1†	141	8.3 ± 2.1†	94	8.4 ± 2.0†	72	9.3 ± 2.0†

s.d., standard deviation.

† , L- shell length measure in millimetre (mm).

### Relationship between environmental variables and *Lymnaea natalensis*

**FIGURE 4:**
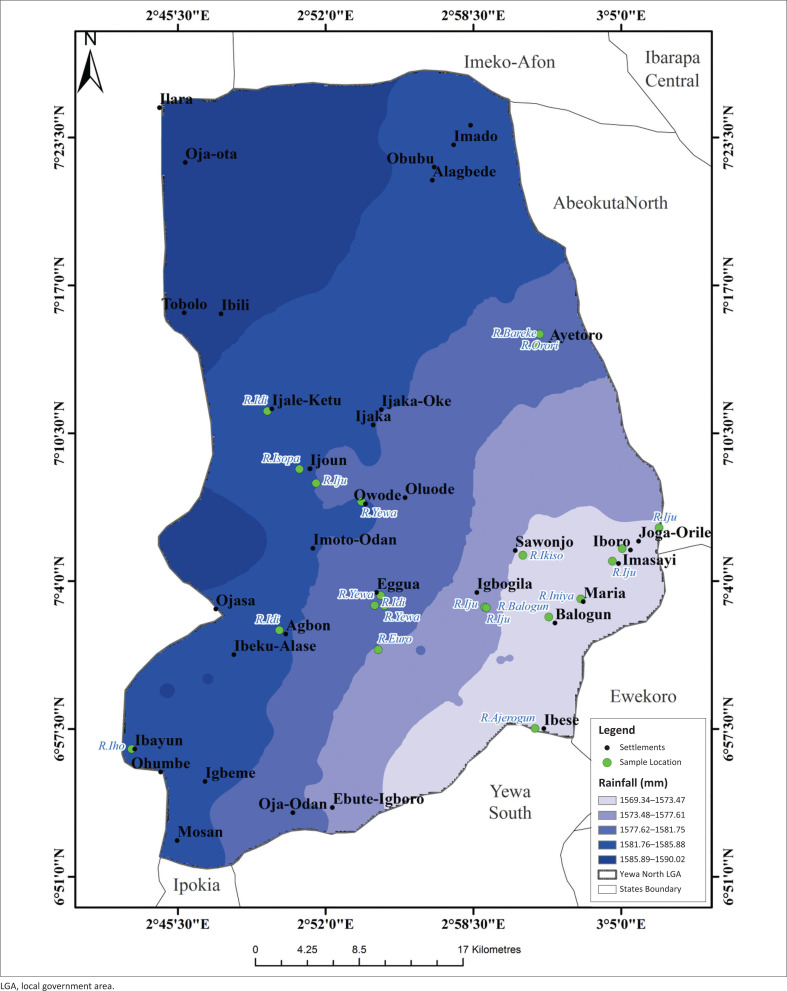
Spatial rainfall map of Yewa North local government area.

**FIGURE 5:**
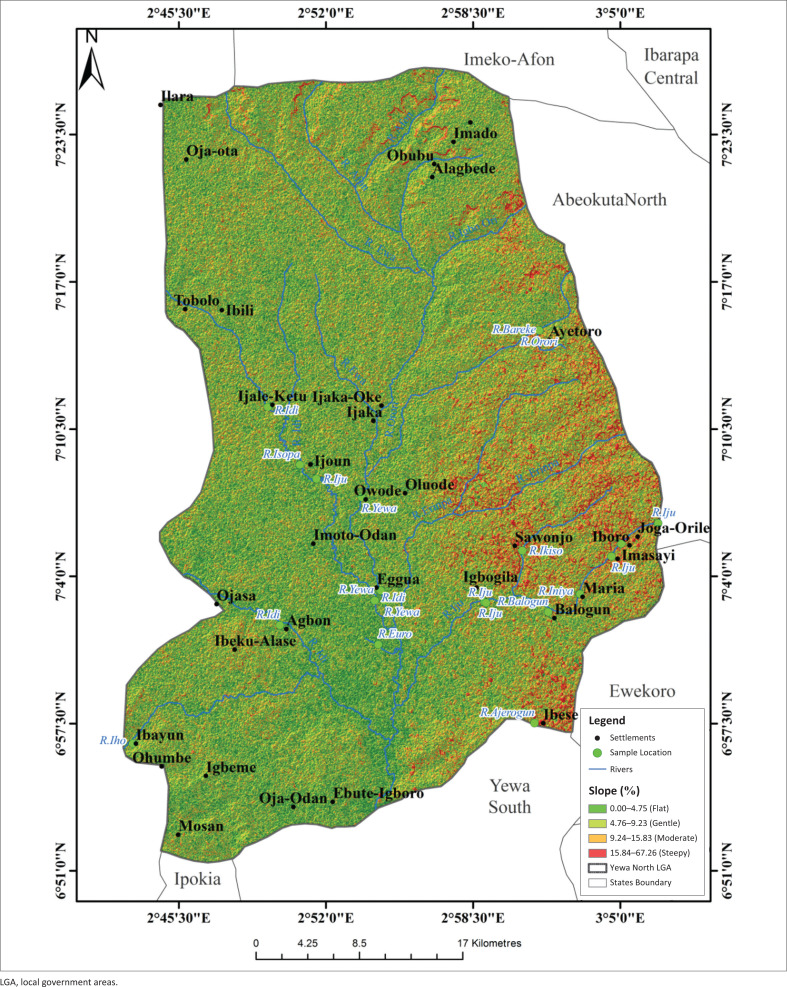
Slope map of Yewa North local government area.

Figure 4 shows the rainfall pattern of YNLGA as captured by satellite imagery. The maximum rainfall was recorded in Tobolo, Oja-Ota and Ibili areas. Ijale-Ketu, Ijaka, Imoto-Odan, Agbon, Ibayun, Alagbede, Ibeku, Ayetoro, Ijoun, Owode, Oja-Odan and Igbeme had relatively medium rainfall. Rainfall had negative relationship with the abundance of *L. natalensis* (*p* < 0.05). The slope of YNLGA (Figure 5) had four different categories, namely flat, gentle, moderate and steep. The middle and western part of YNLGA were flat while the east as well as north-east areas were steep. Ibeku-Alase, Mosan and Ijale-Ketu communities had gentle to moderate slope. A positive relationship occurred between slope and the abundance of *L. natalensis* (*p* < 0.05).

**FIGURE 6:**
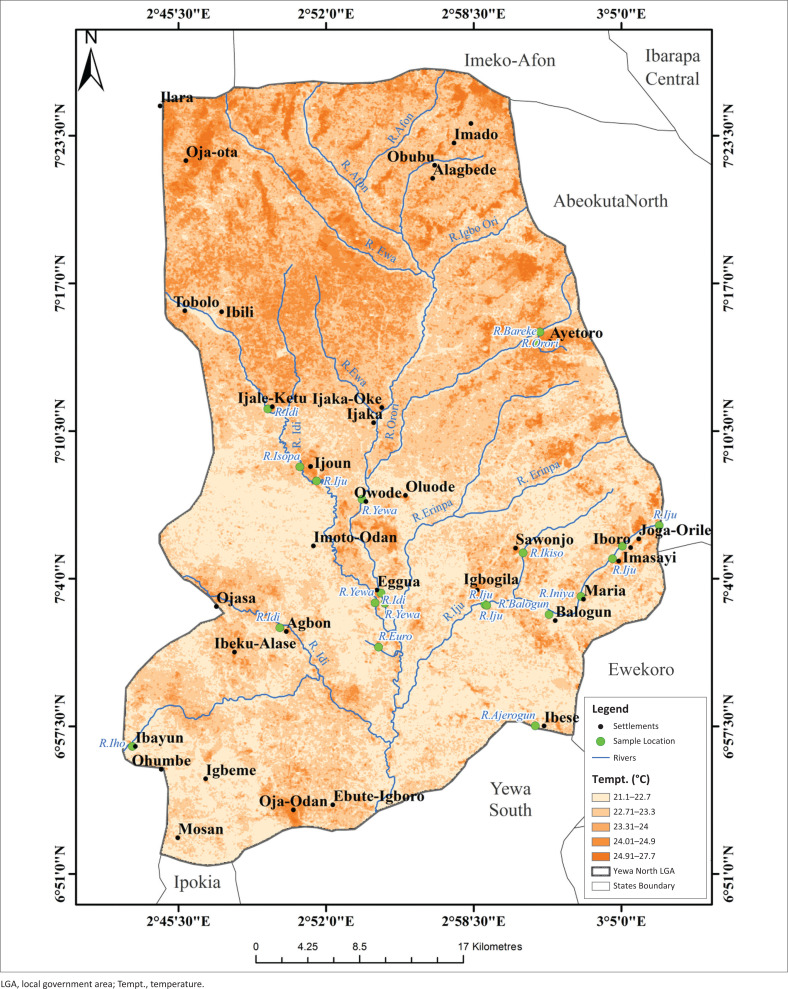
Land surface temperature map of Yewa North local government area.

**FIGURE 7:**
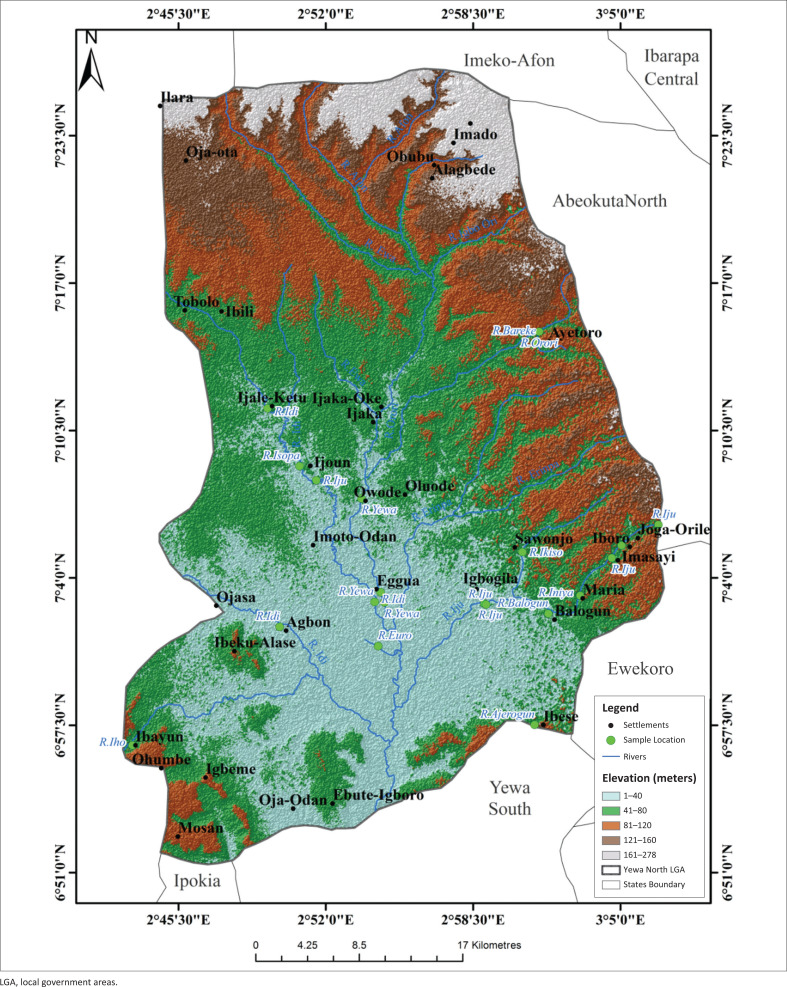
Elevation map of Yewa North local government area.

**FIGURE 8:**
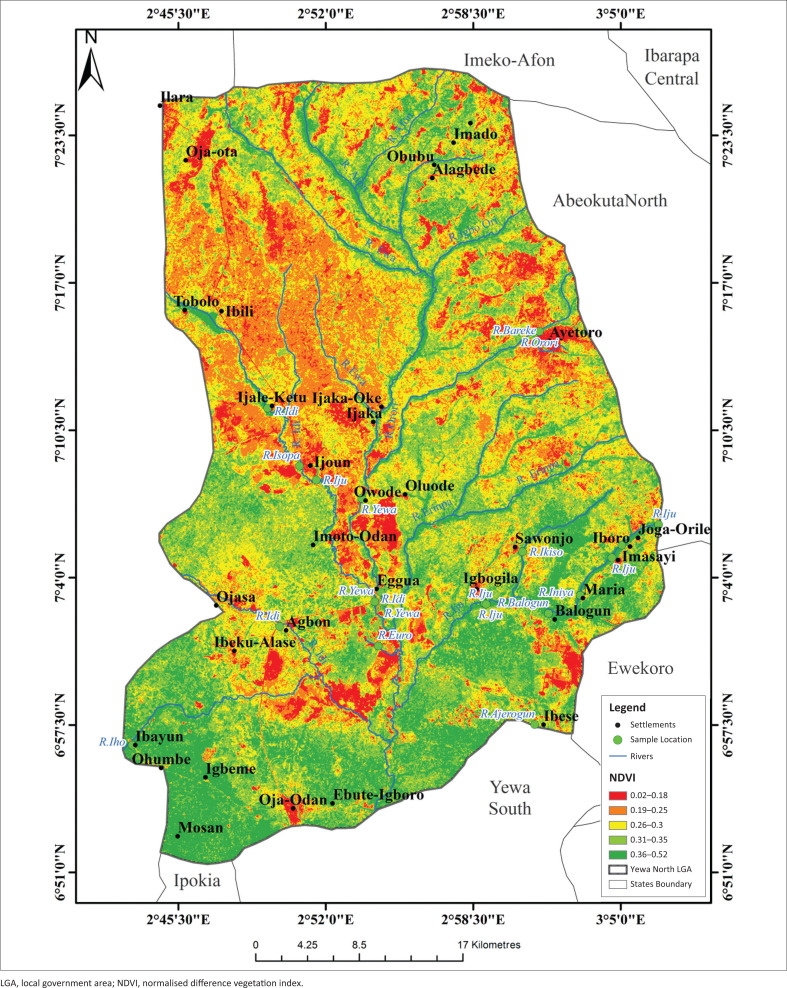
Normalized Difference Vegetation Index map of Yewa North local government area.

The highest LST were recorded in the northern sites (Ayetoro, Igbogila, and Ijoun) with temperature ranges between 24.91 °C and 27.7 °C. Temperatures were lowest in the south, except for Ojo-Odan where LST was between 21.1 °C and 22.7 °C (Figure 6). Land surface temperature had no correlation with the abundance of *L. natalensis*. The highest part of the study area was found in the extreme north of study area with elevation value of between 161 m and 278 m while the least elevated areas were found towards the middle and the southern part of the YNLGA (Figure 7). Areas around Ayetoro, Iboro, Imasayi, Maria, Mosan and Ibayun were moderately elevated with elevation values ranging from 41 m to 120 m. This study shows that elevation had no effect on the abundance of *L. natalensis*. Areas in the southern part of YNLGA had higher NDVI values compared with other part of the study area except in Oja-Odan while the least NDVI values were recorded in the northwestern area. Oja-Ota, Oja-Odan, Ayetoro, Ijoun, Imoto and Ijaka had the highest NDVI values (Figure 8). Normalized Difference Vegetation Index had positive effect on the abundance of *L. natalensis* in the study (*p* < 0.05).

**FIGURE 9:**
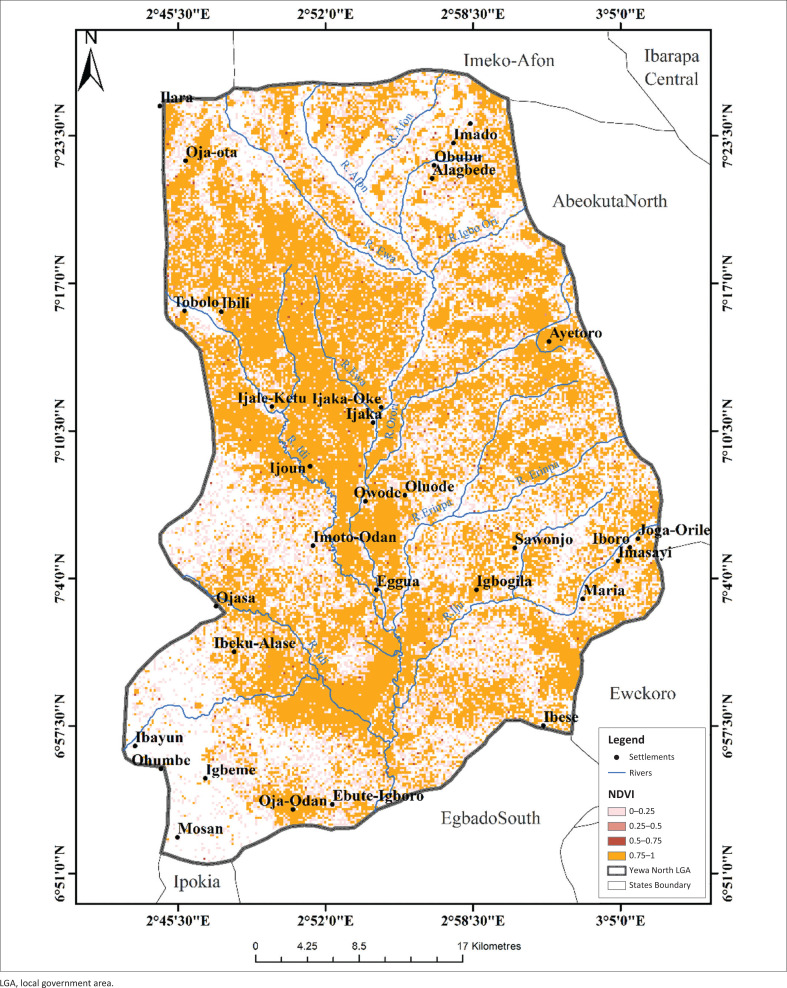
Predictive risk map of *Lymnaea natalensis* habitat.

Predictive risk model showed that most areas in YNLGA were suitable for the survival of *L. natalensis* except some middle belt of study area. The exploratory regression analysis showed that rainfall, NDVI and slope were the three major significant variables important in predicting the spatial distribution of *L. natalensis*. The passing model using the R square and Akaike’s Information Criterion (AICC) identified Apata, Adelabu, Imasayi, Ajagbe, Ijoun, Oja-Odan, Oja-Ota and Ijale-Ketu as major areas where *L. natalensis* can survive. A predictive risk map of *L. natalensis* habitat was created based on the final exploratory regression analysis (Figure 9). High risk areas were mainly located in northwest, while low risk areas were in the southwest.

## Discussion and conclusion

Variations exist in the overall *L. natalensis* abundance between sites and similar variations have been reported in different studies across Africa. The variations occurred as a result of differences between sites in vegetation types (Ofoezie 1999; Olkeba et al. 2020), substratum (Appleton 1974) and the presence or absence of other freshwater snail species (Brown 1994). Another important source of variation between sites is local rainfall, seasonal water flows and water temperature (Woolhouse 1992). From this study, it was apparent that *L. natalensis* populations undergo marked seasonal variation in abundance although patterns differ between sampling sites. This is consistent with other similar studies (Belot, Geerts & Diouf 1993; Ofoezie 1999). Low snail densities during the peak rainy periods have been attributed to the flushing out of snails because of flooding of water bodies (Belot et al. 1993; Coulibaly & Madsen 1990). Most times, at the end of the long rainfall, snail population tends to increase. This has been attributed to the start of oviposition by the adult snails, which aestivated during the dry season (Adie et al. 2015; Webb & Barnwell 1962). As the rain arrives and the river fills up, snails are triggered out of aestivation and repopulate the water bodies. The effect of rainfall on snails depends on the intensity with which the rains fall; published reports on the length of aestivation period do not indicate survival of snails beyond 3−12 months (Leonardo et al. 2020; Sturrock 1993). The pattern of rainfall during this study suggests that heavy downpours of rainfall over a short period of time fill up river bodies, which eventually leads to a reduction in snail populations. This was observed from April to November but there was a break in August at times, where the amount of rainfall reduced. On the other hand, sparse rainfall over a period, from December to March, which has been the pattern in some of the study areas, does not result in a sufficient amount of water that leads to overflow of river boundaries in most of the sampling sites.

Temperature is one of the most important factors affecting performance and abundance of organisms (Angilletta 2009). Organisms that live in water depend strongly on ambient temperature and respond physiologically to temperature variation. While seasonal changes in temperature are highly predictable (Dutta et al. 2018; Kingsolver & Huey 1998; Sørensen & Loeschcke 2002), the unpredictable occurrence of extreme temperatures can have deleterious effect on organisms (Roth, Feichtinger & Hertel et al. 2010; Roux et al. 2010; Wagner et al. 2008). Also, when temperature becomes too extreme, enzymatic function, membrane structure, metabolic rate and oxygen supply can be impaired (Dutta et al. 2018; Philipp & Abele 2010; Pörtner, Langenbuch & Michaelidis 2005). In addition, exposure to high temperature may lead to a higher requirement for micronutrients (e.g., zinc, copper, vitamins) (Askew 1995; Chen et al. 2003; Mnkandla, Basopo & Siwela 2019) as they are involved in response and tolerance mechanisms (Hänsch & Mendel 2009). Absence of relationship between LST and *L. natalensis* was in consonance with other study elsewhere (Kabatereine et al. 2004). Freshwater snail intermediate hosts have definite LST for optimal development. In the transmission of some snail-borne diseases, LST affects the dynamics of some diseases such as the rate of miracidia penetration, cercaria shedding as well as pre-patent infection period (Adekiya et al. 2020; Anderson et al. 1982).

The positive association observed between the NDVI and *L. natalensis* was in consonance with other studies elsewhere (Clements et al. 2008). However, some observed no relationship between NDVI and distribution of other pulmonates (Ekpo & Mafiana 2004). Normalised difference vegetation idex is one of the important vegetation indices and has been used in predicting the habitat of freshwater snails in different areas (Raso et al. 2005). Most times, freshwater snails are usually associated with macrophytes in their habitats. These aquatic plants serve as sources of food and shelter for the snail species; other benefits derived from these aquatic plants include preservation from direct radiation, egg-laying sites, and prevention from wash-off by high water current (Appleton & Madsen 2012).

Areas suitable for snail intermediate hosts of disease organisms depend on indicators provided by pattern analysis. Analyses and inference from similar settings seldom enable prediction of changes in the snail population resulting from ecological transformation caused by anthropogenic activities (Li et al. 2017). The positive association that occurred between slope and *L. natalensis* was in consonance with some findings (Schur et al. 2011; Yang et al. 2009). Highly steep areas are associated with fast flowing water, which usually does not hold water; hence, the possibility of maintaining high population of lymnaeid snails reduces. Steep areas are often used to plant trees, either to serve as wind break or for other commercial purposes; however, this impedes the breeding of the snails. Therefore, as the slope increases, the ability of snail species to survive in such areas decreases (Zhu et al. 2015). This study found that slope in the study areas were within the tolerance range; hence, snail species could survive and populate river bodies.

The method used for infection status in snail species is considered a limitation to this study because no infection was recorded during the study. Use of a more sensitive method for infection in snail species, such as molecular method, could have revealed infections in snail species.

In conclusion, the model produced from this work could be used in modelling other areas where *L. natalensis* could be found, thereby reducing the cost of field epidemiological studies.
